# Does Emotion Regulation Predict Gains in Exercise-Induced Fitness? A Prospective Mixed-Effects Study with Elite Helicopter Pilots

**DOI:** 10.3390/ijerph17114174

**Published:** 2020-06-11

**Authors:** David Cárdenas, Iker Madinabeitia, Francisco Alarcón, José C. Perales

**Affiliations:** 1 Department of Physical Education and Sport, University of Granada, 18071 Granada, Spain; ikermadi@ugr.es; 2 Sport and Health University Research Institute (iMUDS), University of Granada, 18071 Granada, Spain; 3 Department of Didactic General and Specific Training, Faculty of Education, University of Alicante, 03690 Alicante, Spain; f.alarcon@ua.es; 4 Department of Experimental Psychology, University of Granada, 18071 Granada, Spain; jcesar@ugr.es; 5 Mind, Brain, and Behavior Research Center (CIMCYC), University of Granada, 18071 Granada, Spain

**Keywords:** emotion regulation, military, training, physical activity, fitness

## Abstract

Emotion regulation (ER) is a strong predictor of different aspects of mental health and wellbeing. However, only recently has ER been examined in relation to physical activity and its effects on fitness. In the present study, 26 elite helicopter pilots, serving in the Spanish Air Force, were physically trained for 6 months, and their level of fitness (maximum oxygen consumption and time to exhaustion in a treadmill-running test) was assessed before and after that period. Additionally, two indices of emotion regulation (general adaptiveness of ER strategies, as measured by the Emotion Regulation Questionnaire (ERQ), and negative urgency, as measured by the UPPS-P questionnaire) measured at baseline were used as prospective predictors of fitness improvement. After controlling for individual features, baseline fitness, and type of training, better emotion regulation strategies (more cognitive reappraisal plus less expressive suppression) predicted larger fitness gains (*p* = 0.028). Incidental emotion regulation, as measured by the negative urgency index, failed to predict pre–post-fitness changes (*p* = 0.734). These results suggest that fostering emotion regulation skills may improve the effectiveness of fitness training programs.

## 1. Introduction

Emotion regulation (ER) modulates the quality, intensity, and temporal course of experienced emotions [1]. Therefore, ER can be regarded as an umbrella construct entailing the processes through which we control our degree of exposure to emotion-triggering stimuli, direct attention towards (or away from) them, we (re)appraise emotion-laden situations, or inhibit the behavioral expression of such emotions [2]. These processes can operate both incidentally and intentionally, and at any point of the cascading process from emotion elicitation to behavioral expression [3,4]. Additionally, ER can either boost or dampen both positive and negative emotions [5,6] and plays a significant role in social interaction, cognitive functioning, self-control, and mental health and wellbeing [2,7,8].

Recently, Etkin, Büchel, and Gross [9] have proposed a unified model for ER in the broader context of reinforcement-driven decision making. In this model, model-free ER is incidental, operates with little or no intention on the part of the individual, and would be responsible for automatic adjustment of emotion to the ongoing situation (by means of associative activation and deactivation). Model-based ER, however, depends on the intentional use of strategies that the individual has successfully used and perfected by trial-and-error in the past, and its implementation requires the representation of the regulated emotion as a desired state or goal (e.g., reappraisal and diverting attention).

Although altered model-free ER has been hypothesized to underlie a number of self-regulation problems [10,11], model-based ER is more accessible to participants’ awareness and is thus easier to assess by means of self-report tools. In a seminal work, Gross and John [12] developed a questionnaire (the Emotion Regulation Questionnaire (ERQ)) that assesses the dispositional use of two ER strategies: reappraisal, consisting of the cognitive revaluation of the internal or external circumstances that triggered the experienced emotion, and suppression, the effortful inhibition of the observable manifestations of the experienced emotion. Evidence has consistently shown that the regular use of reappraisal predicts better psychological outcomes and wellbeing, whereas frequent use of suppression is associated with self-regulation problems and poorer mental health [12,13].

To date, model-free ER can be assessed only indirectly. Given that, by definition, incidental mechanisms are not fully accessible to self-observation, the alteration of model-free ER has been proposed to be assessed through its manifestation in the form of emotion-driven impulsivity [14]. More specifically, the UPPS-P model of impulsivity [15] proposes its dimension of negative urgency—the tendency to lose control under the influence of strong negative emotional states—as an index of the failure to regulate negative emotions (e.g., irritation, frustration, and anger [16,17,18]). Evidence supporting that proposal stems from studies that have compellingly shown that negative urgency is a key transdiagnostic factor for behavioral disorders in which altered ER plays a well-established etiological role [11,19]. An updated discussion on negative urgency as a proxy to incidental or model-free emotion dysregulation can be found in References [20,21].

More directly related to the aims of the present study, ER is also tightly and bidirectionally linked to exercise. On the one hand, exercise itself works as an overt ER strategy. For instance, it has been shown that exercising before exposure to a stressor reduces the intensity of the ensuing negative emotion [22,23]. Positive acute effects of exercise on mood have also been reported and have been hypothesized to partially depend on exercise-induced release of β-endorphins and endocannabinoids [24,25]. Complementarily, regular exercise has been consistently associated with mood improvement and maintenance [26].

On the other hand, ER strategies also seem to have an impact on exercising. Exercise, when it is performed at high intensity levels, produces reduced effect compared to baseline [26]. Therefore, associative and dissociative cognitive techniques (e.g., self-talk, distraction, visualization, and embodiment) can boost exercise and can facilitate training tolerance by attenuating the less pleasant components of physical effort [27,28,29,30]. In other words, these cognitive techniques can be considered as ER strategies. Actually, it has been proposed that exercising regularly can strengthen ER and can facilitate the generalization of adaptive ER to coping with other stressors unrelated to exercise [22,31].

In view of that, it can by hypothesised that effective ER makes individuals more prone to persevering in training regimes and thus more likely to see their levels of fitness improve. Indeed, there is preliminary evidence of a cross-sectional association between some fitness measures and indices of adaptive ER [32,33,34] as well as of the involvement of shared brain mechanisms in ER and exercise tolerance, such as the thalamus-insula-somatosensory cortex for perceived exertion and the thalamus-amygdala-nucleus accumbens axis for affective responses [35,36,37]. Unfortunately, these studies cannot establish the temporal order of the relationship between ER, voluntary exercise, and fitness. To the authors’ knowledge, no prospective studies on the predictive value of ER scores in relation to subsequent training-induced fitness gains exist (although Hall and Fong [38] proposed a model that can be regarded as closely related to this idea). The aim of this study was to close that gap, (1) assessing emotion regulation indices at baseline, then (2) measuring fitness gains throughout 6 months of physical training, and finally (3) testing whether the size of fitness improvement was prospectively predicted by ER indices.

As noted above, both intentional and incidental ER processes have been linked to self-regulation. For instance, it has been shown that negative urgency leads to more risky behaviors and worse self-control [17]. Moreover, similar problems have been observed when individuals use little reappraisal and, especially, excessive suppression [7,39]. In consequence, and given that there are no a priori theoretical reasons to differentially associate such a potential improvement to intentional or incidental processes, both types of emotion regulation were assessed here. A composite score of (intentional) ER strategies’ adaptiveness (reappraisal minus suppression) was hypothesized to predict larger fitness improvements after training (via superior exercise tolerance or stronger commitment with training), whereas negative urgency was hypothesized to predict a reduction in fitness improvement (measured as a composite measure of VO_2max_ and time to exhaustion in a maximum effort running test).

Importantly, the sample of the present study consisted exclusively of elite helicopter pilots currently serving in the Spanish Army. This population is not widely represented in exercise studies and is interesting in itself. Beyond that, the homogeneity of the sample in terms of age, sociodemographic variables, education, cognitive preservation, and healthy body composition allows for a better control of typical confounders relative to related studies [32,40,41,42].

## 2. Methods

### 2.1. Participants

All participants in the present study took part in a larger project aimed at exploring the role of physical fitness in the psychological and behavioral responses to mental workload in active members of the Spanish Air Force. Results regarding outcome variables not considered in the present study have been presented in previous reports [32,43].

Forty-three male helicopter pilots volunteered for the study; 15 of them were the totality of active Spanish Tigre helicopter pilots when the study started. Participants were randomly assigned to three different 6-month training regimes (endurance training, strengthening (pooled together as the monitored training group for merely descriptive purposes in Table 1; 23 participants), and free training (20 participants; see the Supplementary Materials for details)). Participants who suffered any injury during the training period, making fitness tests impossible, and those who became unreachable after being transferred to a different base were excluded. Twenty-six participants (mean (SD) age = 42.15 (8.37)) went through the whole protocol. Characteristics of the sample are displayed in Table 1.

All participants provided informed consent. The procedure complied with the Helsinki declaration and later amendments (2013) and was approved by the Human Research Ethics Committee of the University of Granada (approval numbers 850 and 12/CEIH2016).

### 2.2. Study Design

Analyses conformed to a correlational, prospective, pre–post design. Namely, initial assessments were used as predictors of change in fitness. As we were not interested in the effectiveness of the specific training methods we used, no control group (with no training) was included in the study.

More specifically, ER scores taken at baseline were used to predict individual pre–post changes in fitness, measured as VO_2max_ and time to exhaustion in a maximal effort running test. The final number of available participants was insufficient to analyze the possible effect of training type—either free or monitored—and fitness level changes were computed regardless of that factor. To prevent any contamination of results, data from all participants were analyzed simultaneously, and training type was treated as a random-effects factor (see the Statistical Analyses section for details), used to model variability in the dependent variable attributable to it but irrelevant for theoretical purposes.

### 2.3. Measures

#### 2.3.1. Fitness Improvement

The endurance fitness level was assessed before and after the training period. An h/p/COSMOS pulsar (Nussdorf-Traunstein, Germany) treadmill was used. The procedure followed in both sessions consisted of a 3-min warm-up at 8 km·h^−1^ and 1% slope. After the warm-up, the treadmill speed was set to 10 km·h^−1^, from which the incremental part of the test started, and every 15 s, the speed was increased 0.25 km·h^−1^ until volitional exhaustion. The test ended with an active recovery period of 5 min in which the participants walked at 4 km·h^−1^ (0° slope). The electrocardiogram (EKG) signal and pulmonary gas (Ultima CardiO_2_; Medical Graphics Corporation, St. Louis, MO, USA) were recorded in the whole session (from warm-up stage to active recovery period). Fitness measures were VO_2max_ (oxygen consumption at exhaustion) and time to exhaustion. Both tests were carried out by physicians from the Andalusian Centre of Sports Medicine.

Two measures of pre–post-training fitness improvement were thus available (∆VO_2max_ and ∆Time-to-exhaustion). In order to reduce the impact of measurement error and their differential sensitivities to different aspects of endurance fitness, and to reduce the number and complexity of further analyses, both measures were combined into a factorial score [44]. After zero-centering and scaling the two differential measures, principal components analysis (PCA) was used to extract a single common factor that explained 73.5% of the variance in the two measures (the factor loading for both individual measures was 0.857). In further analyses, the factorial score will be used as an estimate of pre–post-fitness gain.

#### 2.3.2. Model-Based Emotion Regulation

The Emotion Regulation Questionnaire (ERQ [12]; Spanish version validated by Cabello et al. [45]) is a 10-item scale designed to measure respondents’ tendency to regulate their emotions using cognitive reappraisal (6 items; e.g., “I control my emotions by changing the way I think about the situation I’m in” and “When I am faced with a stressful situation, I make myself think about it in a way that helps me stay calm”; α extracted from the current data = 0.79) and expressive suppression (4 items; e.g., “I keep my emotions to myself” and “I control my emotions by not expressing them”; α extracted from the current data = 0.75). Responses are provided on a Likert-type scale ranging from 1 (strongly disagree) to 7 (strongly agree). The items for each emotion regulation strategy were summed to their total score, with higher ratings indicating greater strategy use.

The two subscales presented an *r* = 0.55 correlation (probably due to the fact that people experiencing more frequent or intense emotions are more frequently in need of regulating them). Therefore, in order to assess the balance between the two strategies and, thus, global adaptiveness of intentional ER a single strategic ER score was computed as the difference between ERQ reappraisal and suppression standardized scores. Indeed, given that most validations have shown the two scores to be orthogonal [46] and, at the same time, to be related with emotional wellbeing in opposite directions [7], the proneness to use reappraisal and not suppression (or vice-versa) can be used to measure individual differences in the adaptiveness of emotion regulation strategies (see also Yoon, Maltby, and Joormann [47]).

#### 2.3.3. Model-Free Emotion Regulation

The Spanish version [48] of the UPPS-P impulsive behavior scale [14] contains 20 items and allows for a 5-dimension assessment of impulsivity although, in this study, only the negative urgency scale (e.g., “When I am upset, I often act without thinking”; α extracted from the current data = 0.68) was used.

## 3. Statistical Analyses and Results

### 3.1. Linear Mixed-Effects (LME) Analyses

The database and code for the analyses described in this section are available without restriction in the Open Science Framework (OSF) website (https://bit.ly/2XNVee4).

The main outcome measure was the factorial pre–post-fitness gain score. This variable was fitted using mixed-effect models (with the lmer function from the nlme R package (version, company, city, state, country)) [49]. All quantitative predictors were also scaled and zero-centered before entering analyses. Comparative model fit was assessed using the Akaike Information Criterion (AIC), the Bayesian Information Criterion (BIC), and χ^2^ tests. Effect sizes were calculated using the Nakagawa–Schielzeth approach [50].

A baseline model (upper row in Table 2) was built with fitness gain as the only output variable; training regime type (free, endurance, and strengthening) as a random-effects factor; and baseline VO_2max_ and baseline time to exhaustion, age, and weight as fixed factors. These baseline-model factors were included as potential confounders, which implies that the contribution of emotion regulation predictors (ERQ/Negative urgency in further models was computed while controlling for such confounders.

In order to test whether ER strategies predicted fitness gains, the combined ERQ strategic ER score was added to the baseline model (ERQ model in Table 2). The contribution of ER strategies to model fit was tested by contrasting the ERQ model against the baseline model. The same procedure was followed to test the effect of negative urgency (contrasting the negative urgency model in Table 2 against the baseline model). This hierarchical test allows to check whether the increase in the proportion of explained variability attributable to the independent variable of interest (either strategic ER or negative urgency), relative to a model without that independent variable, compensates the increase in complexity of the model. As noted above, the inclusion of regime type, baseline fitness level, and weight in the baseline model (and thus also in the ER model) allows to control for the effects of those potential confounders on pre–post-fitness improvements (in a way logically similar to how it is usually done in hierarchical regression analyses).

Models are shown in Table 2. Akaike Information Criterion (AIC) and Bayesian Information Criterion (BIC) values as well as the χ^2^ test show a better fit for the ERQ model than for the baseline model, showing that strategic ER substantially contributes to pre–post-fitness improvement. However, the negative urgency model was clearly not superior to the baseline model, and thus the variability attributable to negative urgency is negligible. In other words, once potential confounders are controlled for, the more adaptive intentional ER strategies, as measured by the ERQ questionnaire but not incidental ER (as measured by the negative urgency UPPS-P index), predicted a larger post-training fitness improvement. The non-standardized coefficient (standard error) for the effect of ER strategies was 0.320 (0.139), with an effect size (using the Nakagawa–Schielzeth approach [50] of R^2^ = 0.175 (CI: 0.467–0.006).

### 3.2. Power Analysis

An a priori power analysis was not feasible in the present study due to strong restrictions regarding participants’ availability (as noted earlier, 15 of the initial 43 participants were the whole active population of Tiger helicopter pilots in Spain). Still, a power analysis was carried out by running Monte Carlo simulations with the best-fitting model using the simr R package and setting the effect size for ER strategies at B = 0.4 (moderate size; 0.4 units of fitness improvement per ER unit, after zero-centring and scaling both variables; standardized β = 0.147). This analysis yielded a 78% power for such an effect.

## 4. Discussion

In keeping with preliminary evidence suggesting cross-sectional associations between emotional wellbeing and different aspects of physical activity and fitness in several populations [32,33,34], the present study is the first to show that the dispositional use of adaptive ER strategies (e.g., more frequent cognitive reappraisal and less frequent expressive suppression) prospectively predicts better outcomes of training regimes.

Despite the fact that previous studies have shown that negative urgency is related to self-regulation problems, no prospective value was found in the present one for this dimension of ER/impulsivity, regarding fitness gains. This discrepancy is probably attributable to the fact that the relationship between negative urgency and poor self-regulation is mainly driven by individuals close to or above the clinical threshold for such problems [51]. Contrarily, in the present study, participants were highly functional and psychologically healthy individuals from the elite military. Among them, it has been observed that using effective strategies to intentionally regulate their emotional states may contribute to the potential effectiveness of training, a possibility that aligns with evidence suggesting that psychological skills are effective at increasing tolerance to exertion. Indeed, such skills (e.g., self-talk, dissociative and associative techniques, and refocusing) overlap in terms of their underlying cognitive processes with reappraisal [27,28,29,30] and can be regarding as incompatible with suppression. (For a discussion on the inverse relationship between suppression and more functional ER strategies, see Navas et al. [16]).

With regard to the specific mechanisms potentially accounting for the link between ER strategies and fitness gains, several possibilities coexist. On the one hand, it is possible that ER skills enhance within-session engagement: That is, ER could facilitate the investment of effort via increased tolerance to its negative emotional effects [52,53]. Tentatively, exercisers who make a more systematic use of reappraisal (and less frequent use of suppression) are probably more prone to use other ER strategies with which reappraisal shares cognitive components and effects on emotional states (see, for example, Reference [54]) that can be effectively implemented while exercising.

On the other hand, related evidence shows that out-of-session emotional associations of physical activity significantly predict the individual proneness to exercising regularly [55]. Once the intention to exercise has been formed, bridging the intention–action gap requires dealing with the affective factors present in all situations of intertemporal decision-making [56]. In other words, when the possibility of exercising is near (and so is having to decide whether to do it or to remain inactive), such a prospect triggers feelings of internal conflict and distinctive affective states that are also subject to modulation if the individual possesses the ER skills necessary to do so. In line with this argument, a study by Mohiyeddini, Pauli and Bauer [57] found that 49% of variance in physical activity was explained by previous intention to exercise but that the inclusion of intention-triggered emotion in the model as a mediating variable increased that percentage to 66%. These results are compatible with more general evidence showing a strong link between ER skills and self-control [58].

Discriminating between these two potential accounts would require testing whether controlling for adherence (either enforcing it via training monitoring or recording it and estimating its mediational effect) modulates the relationship between ER skill and fitness gains. In the present study, the sample was not sufficiently large to estimate potential training type (monitored vs free) × ER interactions, so further research is warranted.

## 5. Limitations and Strengths

The present results must be assessed in light of a number of limitations. First, as acknowledged above, the sample size was not sufficiently large to make strong claims about the possible direct or modulatory effect of training type on fitness gains. Second, exercise adherence was not assessed in free-training participants beyond the fact that they all declared to have remained physically active during the training period and that the maximal oxygen consumption measure showed some pre–post-fitness improvement.

Nevertheless, some of these limitations have been palliated by a number of methodological measures. As noted above, the estimated power assuming a moderate 0.4 regression coefficient for the ER skill measure yields a power level only slightly below the 80% recommendation and well above the average psychological study. The use of a factorial (latent) score for fitness gains reduces measurement error in the dependent variable and thus the probability that such measurement error gives rise to spurious correlations. Additionally, the variance attributable to training type was controlled for by considering that factor as a random intercept and by incorporating age, weight, or baseline fitness measures as fixed-effects covariates did not substantially attenuate the prospective effect of the ER skill measure.

Finally, although the questionnaires employed in this study are well validated across different populations and are widely used in the relevant literature, some reliabilities are below 0.70 and never exceed 0.80 (Alpha coefficients extracted from the current data). These levels are acceptable, but higher reliabilities would have rendered our analyses more powerful to detect effects that can have remained unnoticed here. Moreover, the authors suggest that, as a future direction for research, follow-up measures could be addressed to ensure that improvements are maintained in the mid or long term.

This study might help to better understand the role of emotions in the training process and, in a practical way, encourage fitness coaches to develop more effective sessions that include ER skills training.

## 6. Conclusions

The present study is, to the authors’ knowledge, the first one to explore the role of emotion regulation in training-induced fitness gains. Dispositional proneness to using reappraisal over suppression prospectively predicted larger fitness improvements. Beyond its theoretical implications, this effect is relevant because it has been observed in a scientifically underrepresented population to whom access is often very limited, and because the consequences of military instruction and training programmes are potentially far-reaching (for them and also for civilians). The results suggest that such training programme could be improved if ER skills were explicitly considered. 

## Figures and Tables

**Table 1:** Sample characteristics.

Variable	Training	Mean (Val_max_–Val_min_)	SE	BF_10_
Age (years)	Free	47.85 (55–40)	1.27	**235.14**
	Monitored	36.46 (46–25)	2.06	
Weight (kg)	Free	77.26 (90.7–67)	2.30	0.40
	Monitored	75.73 (88.5–65)	1.90	
Emotion regulation				
Negative urgency	Free	7.62 (11–4)	0.66	0.38
	Monitored	8.00 (14–5)	0.79	
Reappraisal	Free	28.77 (37–14)	1.59	0.61
	Monitored	25.62 (36–12)	2.12	
Suppression	Free	15.62 (21–8)	1.09	0.91
	Monitored	13.15 (19–7)	1.10	
Fitness measures				
VO_2max_ pre (mL/min)	Free	4002.46 (4865–3155)	142.01	0.36
	Monitored	4002.08 (4664–3002)	116.77	
VO_2max_ post (mL/min)	Free	4161.23 (4890–3247)	130.50	0.56
	Monitored	4331.85 (4885–3894)	85.81	
Time to exh. Pre (s.)	Free	569.62 (735–410)	34.21	0.66
	Monitored	622.00 (800–490)	22.33	
Time to exh. Post (s.)	Free	569.62 (705–450)	23.10	1.38
	Monitored	625.85 (804–566)	17.58	

Note: Val_max_–Val_min_: maximum and minimum observed values of each variable; SE: standard error; BF_10_: Bayes factor in favor of the alternative hypothesis; mL/min: milliliters/minutes; s: seconds; exh.: exhaustion. BFs substantially supporting the alternative (BF > 3) or the null (BF < 1/3) are marked in bold.

**Table 2:** Unstandardized estimates and confidence intervals for model parameters, explained variance, and goodness-of-fit indices in all fitted models.

Parameter	Baseline Model		ERQ Model		Negative Urgency Model	
	B	CI	B	CI	B	CI
Fixed Part						
Intercept	−0.00	−0.28–0.28	−0.00	−0.26–0.26	−0.00	−0.28–0.28
Baseline VO_2max_	−0.54	−0.99–−0.09	−0.44	−0.86–−0.02	−0.56	−1.03–−0.10
Baseline time to exh.	−0.11	−0.60–0.39	−0.15	−0.60–0.30	−0.08	−0.59–0.43
Age	−0.16	−0.48–0.15	−0.19	−0.47–0.10	−0.16	−0.47–0.16
Weight	0.35	−0.07–0.76	0.33	−0.05–0.71	0.37	−0.06–0.80
ERQ score			**0.32**	**0.05–0.59**		
Negative urgency					−0.05	−0.36–0.25
Random part						
σ^2^	0.547		0.454		0.545	
τ_00_	0.000		0.000		0.000	
R^2^/Ω_0_^2^	0.431/ 0.431		0.527/ 0.527		0.434/ 0.434	
AIC	72.105		**69.274**		73.989	
BIC	80.911		**79.339**		84.054	
χ^2^ test			**4.830 (*p* = 0.028)**		0.115 (*p* = 0.734)	

Note: B: unstandardized regression coefficients; CI: 95% Confidence intervals around regression coefficients; *p*: significance of χ^2^ tests for the Emotion Regulation Questionnaire (ERQ) and negative urgency models against the baseline model; σ^2^: error term; τ_00_: variance of random intercept; R^2^/Ω_0_^2^: biased and unbiased estimates of the proportion of variance explained by the full model. AIC: Akaike Information Criterion: BIC: Bayesian Information Criterion. Bold fonts show information of the best model.

## Supplementary Materials

The following are available online at https://www.mdpi.com/1660-4601/17/11/4174/s1.
