# Supplementary material for: Does Emotion Regulation Predict Gains in Exercise-Induced Fitness? A Prospective Mixed-Effects Study with Elite Helicopter Pilots
Source: Int J Environ Res Public Health. 2020 Jun 11;17(11):4174. doi: 10.3390/ijerph17114174 (PMC7312943; doi:10.3390/ijerph17114174)
Supplement: Supplementary file 1 [file ijerph-17-04174-s001.pdf]

**Supplementary Material: Description of Training Regimes**

The project's original design included three groups differing in their training programmes (with the aim of testing the effect of different fitness components on the psychological and behavioural response to mental workload, an objective not directly related to the aims of the present study). However, the number of available participants was insufficient to complete the groups, so training type was included in statistical analyses as a random-effects factor. For the purposes of the present study, this composition indirectly allows the sample to be representative of the mixture of voluntary and mandatory physical activity in the population of reference.

Endurance training participants ($n$ = 8 at the end of the protocol, $n$ = 13 at the beginning) followed a high intensity interval training (HIIT) method, in three sessions per week. The training total time per week was 120 minutes (weeks 1 and 2) or 150 minutes (weeks 3 to 23). Intensity was controlled by using POLAR rs800cx pulsometers. Across sessions, intensity (in within-session high intensity intervals) was progressively increased from 70% to 95% of heart rate reserve.

Strength training ($n$ = 5 at the end of the protocol, $n$ = 10 at the beginning of the study) was programmed by using maximum repetition (MR) to control intensity. Participants in this subgroup trained all muscle groups in a distributed fashion, in three sessions per week over 23 weeks. Training under the 50–70% 1-MR maximum intensity was considered as moderate, and in the 70–90% 1-MR as vigorous. Every four weeks, maximum intensity was readjusted for the whole group.

Free-training participants ($n$ = 13 at the end of the protocol, $n$ = 20 at the beginning of the study) were instructed to freely engage in moderate or vigorous physical activity during the same 23-week period. Prior to that, all participants were physically active and were instructed to – at least – maintain the same level of activity. After the pre-post period, all participants declared to have regularly trained during the whole period. Beyond that, physical activity was not monitored.
